# Specific detection of tau seeding activity in Alzheimer’s disease using rationally designed biosensor cells

**DOI:** 10.1186/s13024-023-00643-2

**Published:** 2023-08-08

**Authors:** Aurelien Lathuiliere, Youhwa Jo, Romain Perbet, Cameron Donahue, Caitlin Commins, Noé Quittot, Zhanyun Fan, Rachel E. Bennett, Bradley T. Hyman

**Affiliations:** 1 Department of Neurology, Massachusetts General Hospital/Harvard Medical School, 114 16Th Street, Charlestown, MA 02129 USA; 2 Harvard Medical School, Boston, MA USA; 3 Memory Center, Department of Rehabilitation and Geriatrics, Geneva University Hospital and University of Geneva, Geneva, Switzerland

**Keywords:** Alzheimer’s disease, Tau, Seeding, Prion-like propagation, Biosensor cells

## Abstract

**Background:**

The prion-like propagation of tau in neurodegenerative disorders implies that misfolded pathological tau can recruit the normal protein and template its aggregation. Here, we report the methods for the development of sensitive biosensor cell lines for the detection of tau seeding activity.

**Results:**

We performed the rational design of novel tau probes based on the current structural knowledge of pathological tau aggregates in Alzheimer’s disease. We generated Förster resonance energy transfer (FRET)-based biosensor stable cell lines and characterized their sensitivity, specificity, and overall ability to detect bioactive tau in human samples. As compared to the reference biosensor line, the optimized probe design resulted in an increased efficiency in the detection of tau seeding. The increased sensitivity allowed for the detection of lower amount of tau seeding competency in human brain samples, while preserving specificity for tau seeds found in Alzheimer’s disease.

**Conclusions:**

This next generation of FRET-based biosensor cells is a novel tool to study tau seeding activity in Alzheimer’s disease human samples, especially in samples with low levels of seeding activity, which may help studying early tau-related pathological events.

**Supplementary Information:**

The online version contains supplementary material available at 10.1186/s13024-023-00643-2.

## Background

The brain accumulation of the microtubule associated protein tau (MAPT) into insoluble aggregates is a major neuropathological feature of Alzheimer’s disease (AD) and other neurodegenerative disorders referred to as tauopathies. Tau is an intrinsically disordered protein normally present in the axonal compartment of neurons where it plays a role in the stabilization of microtubules. In AD, hyperphosphorylated forms of tau accumulate in the cytoplasm and aggregate into paired helical filaments (PHFs) that are beta-pleated sheet filamentous structures [1, 2]. The stereotypical Braak staging of AD tau pathology from the entorhinal cortex, to hippocampus, and later to limbic regions [3], correlates with the loss of neurons and the course of clinical symptoms [4–8]. The spreading of tau aggregates across interconnected anatomical regions is supported by a strong body of evidence. For example, transgenic animals overexpressing P301L mutant tau in layer II of the entorhinal cortex develop human tau aggregates in the dentate gyrus, templating endogenous mouse tau in the process [9]. The capacity of misfolded tau aggregates to recruit normal tau in a “prion-like” mechanism is called seeding and has been replicated in numerous experimental models [10–13]. The role of seeding in the propagation of the human pathology is supported by the detection of seeding-competent tau (also referred to as bioactive tau) in brain regions containing overt neurofibrillary tangle pathology but also in subsequent regions that are free of overt pathological changes, further along the Braak pathway [14]. Tau seeding propensity in postmortem brains correlates with the rate of clinical progression, confirming the fundamental role of this phenomenon in AD [15, 16].

The study of seeding mechanisms in AD pathology was greatly accelerated by the development of a tau biosensor cell line based on Förster resonance energy transfer (FRET) [12]. This stable human embryonic kidney (HEK 293 T) cell line expresses probes that are a P301S pro-aggregating mutant of 4R tau repeat domain (RD, amino acids 244–372) fused to either a cyan fluorescent protein (CFP) or yellow fluorescent protein (YFP). The addition of AD brain-derived seeding-competent tau material or preformed tau fibrils induces the aggregation of this fluorescent probes and subsequent FRET signal. The FRET signal can be quantified by flow cytometry 24 h after incubation with tau seeds. To increase the sensitivity of seed detection, liposomes are mixed with seeding-competent material to transduce seeds into the cells [17]. In the absence of liposomes, the biosensor cells can be used to study the mechanisms of seed uptake [18, 19]. Tau biosensor cells have been used to specifically amplify and quantify minute amounts of bioactive tau from various AD samples including brain extracts using various purification methods, postmortem cerebrospinal fluid (CSF), antemortem lumbar CSF or brain-derived extracellular vesicles [20–24].

Further analysis of tau lesions in AD and other tauopathies reveal differences in both post-translational modifications observed [25, 26] as well as in structure of the fibrils, as defined by cryo-electron microscopy technology. While AD, primary age-related tauopathy, and the rare autosomal dominant Familial Danish or British dementia share the same core structure of tau fibrils, other tauopathies including progressive supranuclear palsy (PSP), cortico basal degeneration (CBD), Pick’s disease (PiD) or chronic traumatic encephalopathy (CTE) have specific structural signatures of tau core aggregates [27]. Although the specific pathological role of tau propagation and templated aggregation is still under investigation in non-AD tauopathies, the existence of tau seeding activity has been demonstrated in different experimental systems, including injection of human brain extracts in transgenic animals [28], or real-time quaking induced conversion (RT-QuIC) [13, 29, 30], for each tauopathy. These findings suggest that specific tau strains or conformers may explain the specific phenotypes of tauopathies. We hypothesized that, given the structural insights noted above, biosensor cells could be designed to have a preference for one class of tau structures compared to others. Here, we describe the methods for the development of a novel tau biosensor cell line with a preference for AD tau.

## Results

### Design of the tau RD FRET probe affects sensitivity

**Fig. 1:**
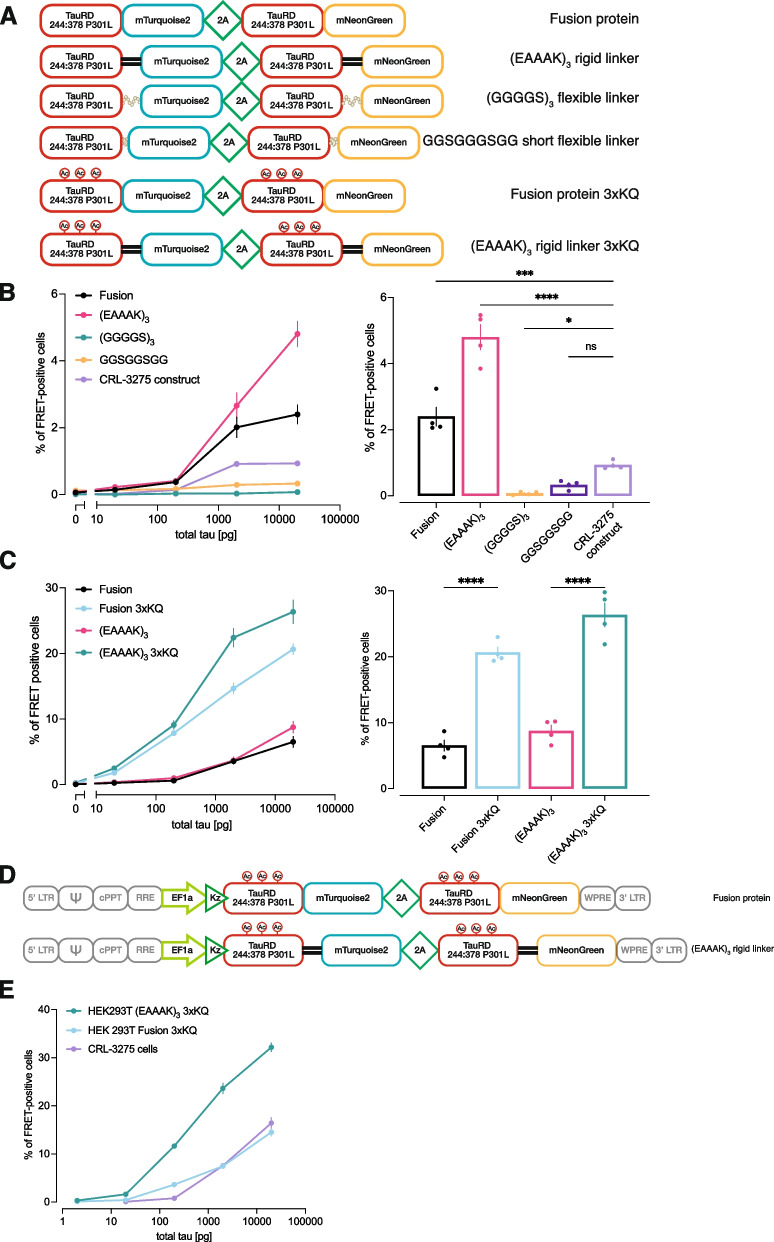
Biosensor probe design affects detection of bioactive tau seeds. **A**. Schematic of the various tau probes that were designed. Tau sequence (244:378) consisted in the repeat domain and amino acids included in the core of AD tau filaments. Variations in the linker sequence composition between tau sequence and fluorescent reporter were introduced in construct. 3 KQ mutations were also introduced to mimic posttranslational modifications. **B**. Tau seeding was quantified by flow cytometry on cells transiently transfected with constructs containing variations in the linker sequence and exposed to serial dilutions of AD brain lysate (left panel). The right panel represents the data extracted for a tau concentration of 20 ng..**C**. Similar experiment performed comparing the effect of 3xKQ modifications on tau sequence. **D**. Schematic of the lentiviral vectors that were produced to generate stable cell lines. **E**. Stable cell populations generated by lentiviral transduction were compared to the original biosensor line in a flow-cytometry based seeding assay. Each point represents mean of 4 biological replicates. Error bars at standard error of the mean

To optimize the expression level and efficacy to detect FRET signal after aggregation of the probe, several modifications were implemented in the probe design. The DNA sequence was based on a single construct comprising a T2A self-cleaving peptide to maintain close to equimolar expression of the two fluorescently labeled tau molecules [31]. The tau sequence from amino acids 244 to 378 covered the repeat domain of tau and additional amino acids that are part of the core structure of cryo electron microscopy (EM) defined AD tau filaments [32]. Modified turquoise 2 (mTurquoise2) and modified neon green (mNeonGreen) were selected respectively as FRET donor and acceptor based on high predicted energy transfer [33, 34]. Because the DNA sequence was very analogous on both sides of the 2A peptide, codon optimization was performed to avoid recombination during DNA integration in host cell genome [35]. The efficiency of FRET energy transfer is inversely correlated to the distance between the fluorophores. We postulated that linker composition and conformation may impact the FRET efficiency. Therefore, we have compared constructs with linkers that have varying conformation and flexibility [36]. Because tau acetylation and/or ubiquitylation at residues 311, 317 and 321 are observed in human AD, and have been proposed to drive assembly into paired helical filaments [26, 37], we mutated lysines at those sites to glutamines to mimic acetylation (3xKQ mutant) (Fig. 1A).

**Fig. 2:**
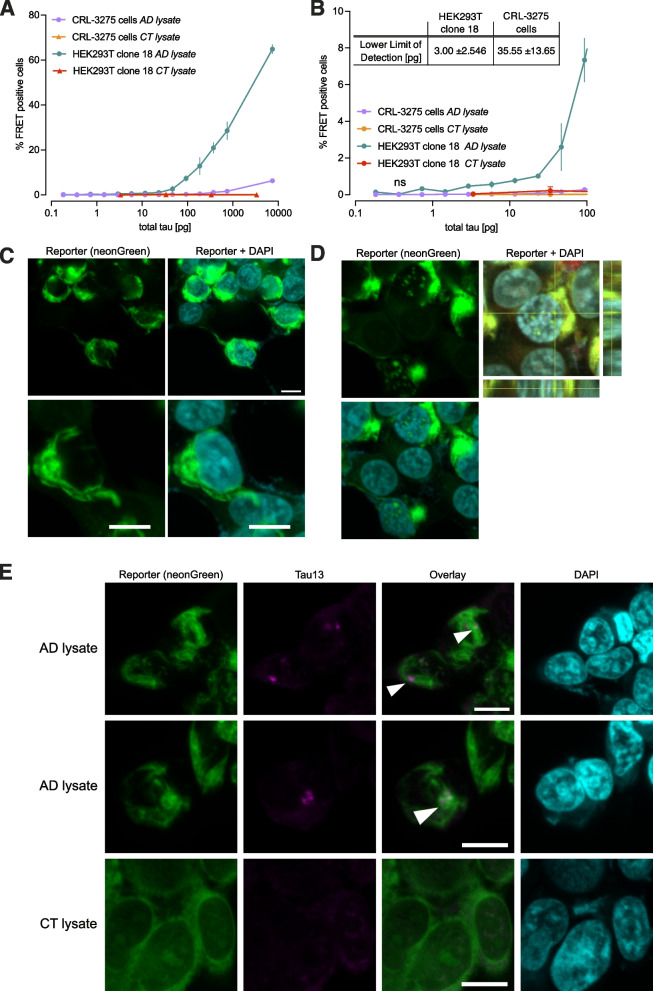
Novel biosensor HEK293T clone 18 demonstrates increased sensitivity in amplifying bioactive tau seeds. **A**. The selected biosensor clone was compared to the original tau biosensor cell line in a flow-cytometry based seeding assay after exposure to serial dilutions of total tau in human AD brain lysate or control brain lysate. **B**. Expansion of A in the lower tau concentration range. Each point represents mean of 4 biological replicates. Error bars at standard error of the mean. The table summarize the calculated lower limit of detection. Values are means of 2 independent experiments ± standard deviation. **C**. Fluorescence imaging of the selected biosensor cell clone 24 h after seeding with AD brain lysate, depicting representative fibrillar structure of aggregated tau probe. Lower panels depict the same condition with higher magnification. **D**. Representative image of tau nuclear speckles detected after seeding in new biosensor cell clone. **E**. Detection of brain-derived human tau-13 positive inclusions (arrowheads) embedded in fibrillar tau probe aggregates 24 h after seeding. Scale bar is 10 μm

To compare the efficiency of different tau probes in detecting human brain derived seeding-competent tau, HEK293T cells were transiently transfected with the various constructs and exposed to serial dilutions of human brain homogenates for 24 h. As a reference, the CFP and YFP tau RD constructs used to generate the original CRL-3275 biosensor line were transferred into a single plasmid containing the 2A peptide [12]. This tau probe includes a 21 random amino acid linker (YPYGILQSTVPRARDPPVATAV). The use of flexible linkers generated lower FRET signal as compared to the original construct, while the fusion protein and 15 amino acid (EAAAK)_3_ rigid linker yielded higher FRET efficiencies when cells were exposed to serial dilutions of tau (Fig. 1B). The pseudoacetylation increased the number of FRET-positive cells by approximately threefold, suggesting that pseudoacetylated tau probes have a higher propensity for human AD brain tau induced aggregation (Fig. 1C).The values of area under the curves are provided in Supplementary Table 1. Based on these findings, lentiviral vectors encoding the pseudoacetylated tau probes were produced (Fig. 1D) and used to generate stable HEK293T cell lines. As compared to the original biosensor line, the stable pseudoacetylated (EAAAK)_3_ expressing cell population demonstrated a ~ tenfold enhanced, robust ability to detect seed competent tau in human AD brain homogenate (Fig. 1E; Fig. 2).

### Novel biosensor HEK293T clone 18 sensitively amplifies bioactive tau seeds into insoluble aggregates

From the most promising stable cell population, 52 individual clones were isolated. All of them were tested in flow cytometry-based seeding assays (data not shown). The clone with the highest signal to noise ratio was selected for further characterization. As compared to the original biosensor cell line, this HEK293T clone (#18) demonstrated high sensitivity in detecting bioactive tau seeds when serial dilutions covering five orders of magnitude in tau concentrations were applied to the cells (Fig. 2A). The calculated lower limit of detection for the clone 18 was 3 pg, more than 10 times lower than the original biosensor cell line (Fig. 2B). To characterize the morphological features of the tau probe aggregates after seeding, clone 18 was imaged on a confocal microscope after seeding with AD or control brain lysate. The fluorescent tau probe aggregates were frequently taking the shape of fibrillar structures, very similar to pathological tau aggregates (Fig. 2C). Notably, we found that aggregates not only accumulated in the cytoplasm, but also in the nucleus, as described previously (Fig. 2D) [38]. Immunostaining with an antibody that does not recognize the probe was performed, using the N-terminal tau-13 antibody. After seeding with AD brain lysate, tau-13-positive inclusions entrapped within the probe aggregates were detected (arrowheads, Fig. 2E). These punctates likely represent seed-competent material.

**Fig. 3:**
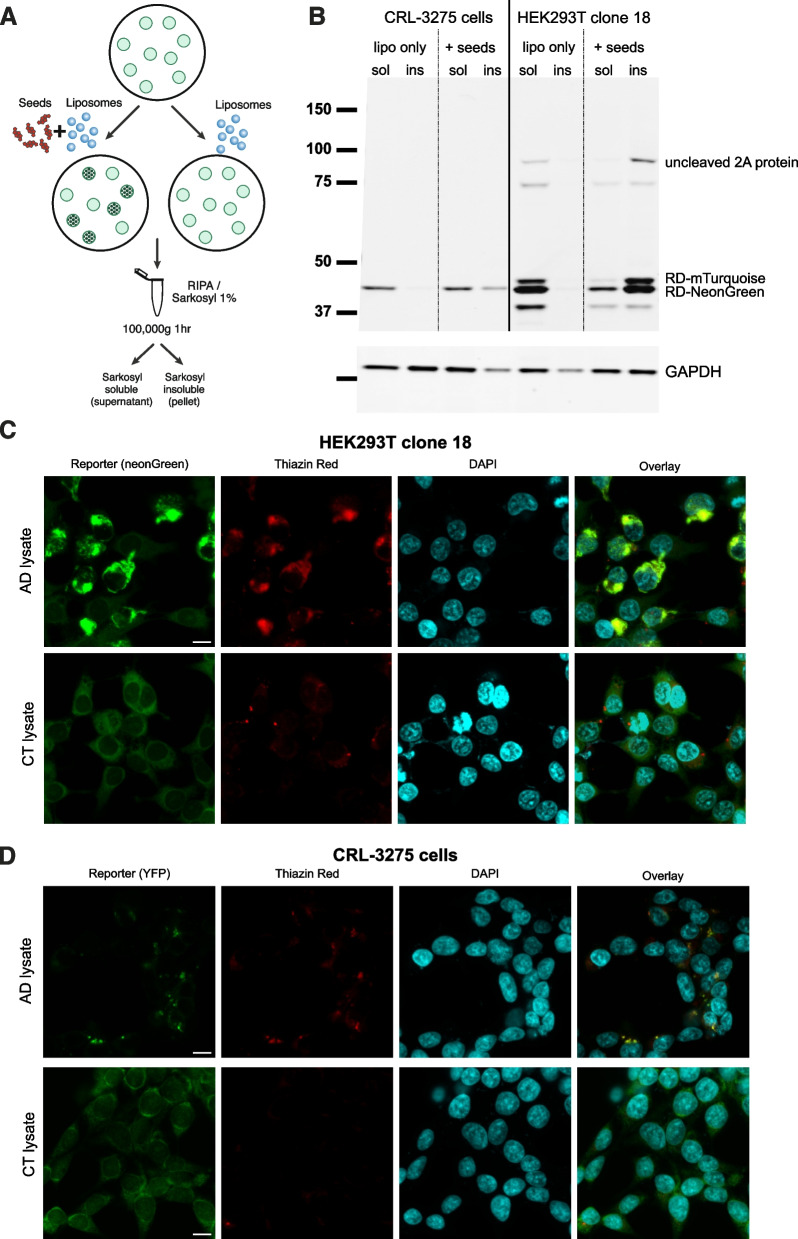
Tau probe in biosensor cells aggregates in insoluble beta pleated structures after seeding. **A**. Schematic of insoluble and soluble tau extraction protocol after seeding. **B**. Western blot comparing tau probe expression in the original biosensor cell line and novel biosensor HEK293T clone 18 after seeding in soluble and sarkosyl insoluble fractions. An antibody recognizing the repeat domain of tau was used as a primary antibody. GAPDH was used as a loading control. Uncropped images of western blot membranes are displayed in Supplementary Fig. 2. Thiazine red staining was performed to detect beta-pleated conformation 24 h after seeding with AD brain lysate or control brain lysate in HEK293T novel biosensor clone (**C**) and original biosensor line **D**. Scale bar is 10 μm

The state of aggregation of the probe after seeding was further analyzed. Proteins were extracted from seeded cells in 1% sarkosyl and run on a western blot using a detection antibody that binds to the tau RD (Fig. 3A). As compared to the CRL-3275 cells, the basal expression level of the FRET probe in HEK293T clone 18 was substantially higher (approximately sixfold increase, Supplementary Figure Fig. 1D). Moreover, after seeding with AD brain derived material, most of the probe accumulated into the sarkosyl insoluble fraction unlike the original cell line. The western blot also revealed the presence of small amount of uncleaved polypeptide that also accumulated in the insoluble fraction. Using this extraction protocol, we found that glyceraldehyde-3-phosphate dehydrogenase (GAPDH), our loading control, was also detected in the sarkosyl insoluble fraction in accordance with other studies [39, 40]. In HEK293T clone 18, the western blot revealed an additional band around 40 kDa which is probably the result of a cleavage within the fluorescent protein as it is still detected with the tau 316–355 antibody. Next, the formation of beta-pleated sheet secondary structure of the probe was assessed using thiazine red staining. Thiazine red is a fluorescent dye that has high affinity for beta pleated fibrillar structures and that stains mature tau aggregates in the AD brain [41]. After seeding with AD brain lysate, the HEK293T new biosensor exhibited a robust thiazine red signal that colocalized with aggregated fluorescent reporter (Fig. 3C). In the original CRL-3275 cell line, the thiazine red positivity was also observed but the size of aggregates was much smaller (Fig. 3D).

### Novel biosensor HEK293T clone 18 detects bioactive tau seeds from the Alzheimer’s disease brain with increased sensitivity and specificity

**Fig. 4:**
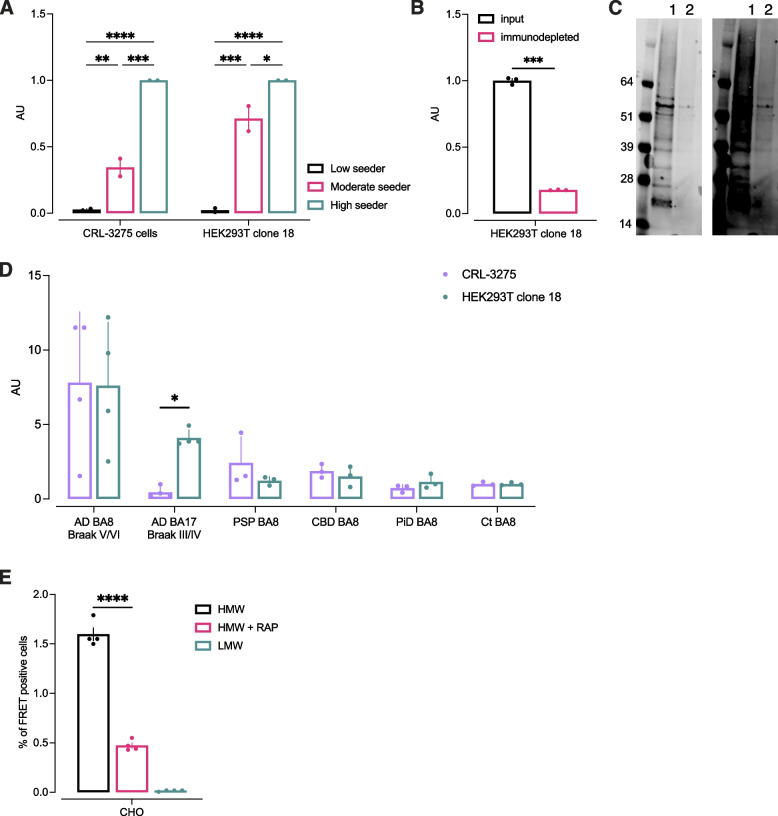
Novel biosensor HEK293T clone 18 amplifies bioactive tau seeds with high sensitivity and specificity. **A**. Brain extracts from AD cases extensively characterized in Dujardin et al. [15], considered as low, moderate, and high seeing activity were tested in flow cytometry-based seeding assays in both the novel and original biosensor lines. Signal was normalized on the high seeder to demonstrate a similar pattern in both lines. Bars represent mean of 2 individual independent experiments. **B**. The specificity of the new tau biosensor clone was confirmed after immunodepleting of tau in AD brain homogenate. Tau was immunodepleted from brain lysate and both input and depleted material were tested in flow-cytometry based seeding assay. Immunodepletion was confirmed by western blot using a polyclonal tau antibody as detection (**C**): lane 1 is input, lane 2 is immunodepleted sample. Membrane shown on the right is a long exposure time showing a smear corresponding to High molecular weight (HMW) tau. **D**. Comparison of tau seeding activity in both biosensor lines across various tauopathies (each point represents the mean of 3 technical replicates of one case, error bars represent the standard deviation). **E**. A stable CHO clone overexpressing the biosensor construct was incubated with AD brain-derived HMW seeding competent tau or low molecular weight (LMW) tau in absence of liposomes for 48 h and uptake and amplification of seeds was quantified by flow cytometry. The addition or 1 μM RAP, a known LRP-1 inhibitor decreased seeding

We have recently demonstrated that some of the variability in the clinical progression of AD correlates with tau seeding activity, as measured by the original biosensor cell line (CRL-3275) in the postmortem AD brain [15]. To investigate whether the novel biosensor HEK293T clone18 detects similar pathological tau species, AD cases previously shown to harbor high, moderate, and low seeding activity were tested in parallel in seeding assays with both cell lines. After normalization, the quantification of seeding activity provided the same pattern in both cell lines, suggesting that the novel biosensor line detects similar clinically relevant tau species (Fig. 4A).

In AD brain homogenate, multiple factors may drive the aggregation of the tau probe. To assess the probe specificity for tau seeds, an immunodepletion was performed. Tau was immunoprecipitated in the human brain lysate using an antibody targeting the mid-region. The starting material and immunodepleted samples were run in the seeding assay using the novel biosensor. In the depleted material, the seeding signal was decreased by 82.3% (Fig. 4B). Samples were analyzed by western blot to evaluate tau amount (Fig. 4C). A smear was found with the starting material confirming the presence of heavily modified and aggregated forms of tau in the AD brain. Some residual bands were present in the immunodepleted sample suggesting that the immunoprecipitation was incomplete, explaining why some residual signal was found in the seeding assay.

Next, to compare the sensitivity and specificity for various tau seeds, brain lysates from AD, progressive supranuclear palsy (PSP), cortico basal degeneration (CBD), Pick’s disease (PiD), and control cases were tested in the original and novel biosensor cells (Fig. 4D). For all cases, the input was normalized on total tau content measured by western blot (50 ng). As expected, when we used brain lysates from a region with overt tau pathology where high seeding activity was expected such as the frontal cortex (Brodmann area 8) in late Braak stages (V/VI), the signal was comparable between lines. Moreover, the novel biosensor line was able to consistently detect seeding activity even in AD lysates from the primary visual cortex (Brodmann area 17) that should be free of tau pathology to qualify for Braak stage III or IV.

The CRL-3275 cells are able to detect tau from non-AD human tauopathies, but appear to have a clear preference for AD related tau “strains” (Fig. 4D). We hypothesized that, by both enhancing FRET efficiency with improved fluorophores and optimized linkers, while at the same time choosing both the domain that closely matches AD fibril cores and by introducing AD-related posttranslational changes at specific lysine residues, we might be able to engineer a more sensitive probe that still maintained its preference for AD related seeds. We tested samples from PiD, PSP, CBD, and controls (Fig. 4D) and found that clone 18 indeed did not show enhanced sensitivity for non-AD tau, since the signal was observed to remain low in other tauopathies (despite matching for equivalent amounts of tau added to the assay).

In AD, tau propagates from one cell to another across interconnected brain regions. The LDL receptor related protein 1 (LRP1) is an endocytic receptor for both monomeric and pathological tau and mediates tau uptake and also seeding [19, 42]. In the absence of liposomes to permeabilize the cell membrane, the original biosensor propagates seeds with low efficiency, possibly related to the relative low levels of LRP1 present in HEK cells. The increased sensitivity of the reporter in the engineered line shows a 10 time increase in FRET signal after incubation with brain derived high molecular weight (HMW) tau preparation as compared to the original line. Interestingly, even though HEK293 cells express low levels of LRP1 [19] the seeding was reduced when HMW was co-incubated with 1 μM RAP, a known antagonist of LRP1 (Supplementary Fig. 2A), suggesting that the enhanced sensitivity observed was not due to an increase in tau uptake, but to a more sensitive reporter system. Since Chinese Hamster Ovary (CHO) cells express higher levels of LRP1 [19], we engineered a clonal cell line overexpressing the same tau biosensor probe. When this stable CHO clone was incubated with HMW tau in the absence of liposomes, seeding was detected. In the presence of RAP, seeding was decreased by two thirds, suggesting that most of tau uptake was mediated by LRP1 (Fig. 4E). This experiment confirmed that such lentiviral-based optimized reporter construct can be used not only to study the templated seeding but also the mechanisms of extracellular tau uptake.

## Discussion

FRET-based tau biosensor cell lines are an efficient method to quantify seeding capacity of tau species in biological samples. Here, we describe a novel AD tau biosensor line with improved sensitivity, using the recently acquired knowledge of the structural features of tau aggregates in AD. We developed tau FRET probes based on a single DNA construct using the T2A self-cleaving peptide. This construct allowed for the efficient overexpression of the tau reporter using a single lentiviral construct. Unlike previous attempts to increase the expression of tau probe in biosensor cells [43], this strategy resulted in a dramatic improvement in the expression of the probe as compared to the original biosensor line. As previously reported by another study that used different fluorescent reporters, the optimization of the fluorescent protein pair allowed for the generation of a robust FRET signal across a wide dynamic range [44].

The tau fragment sequence was selected to include the complete amino acid sequence of the AD core aggregates. Using transient transfection in HEK 293 T cells, we compared multiple linker sequences and found that a 15 amino-acid rigid linker provided an optimized FRET signal measured by flow cytometry. Like the original biosensor line, the novel tau probe included a mutation at position 301. Proline 301 substitutions have been shown to modify the local structure of tau and foster its seeded aggregation [45, 46]. The CRL-3275 line holds a P301S mutation whereas the HEK293T clone 18 holds a P301L mutation. The P301L mutant may potentially explain some of the improved sensitivity of the new cell line. However, both mutants are equally used in the field and previous studies comparing the effects of the two mutants reported inconsistent findings [47, 48].

The cryo-EM structure of tau aggregates in AD has revealed the presence of densities at the level of lysines 317 and 321 [32]. Later, post-translational modification including acetylation and ubiquitination were reported at lysines 311, 317 and 321 in brain derived tau aggregates and were proposed as a mechanism of charge neutralization favoring the stacking of protofilaments into paired helical filaments [26, 37]. The modification of the tau probe mimicking acetylation at residues 311, 317 and 321 resulted in a massive increase of aggregate formation in biosensor cells, suggesting that the modified probe may have higher propensity for aggregation.

Altogether, the multiple optimizations of the tau probe resulted in a cell line with a more than tenfold increase in the sensitivity for tau seeds in AD brain extract. Clone 18 detected significant FRET signal in total tau concentration in the femtomolar equivalent of tau monomers while maintaining specificity for tau seeds as demonstrated by immunodepletion. The increased sensitivity allowed for the detection of tau seeding activity in regions with low expected signal such as the primary visual cortex in Braak III/IV AD cases. In addition, like the original CRL-3275 biosensor cell line, the newly generated clone 18 reporter detected consistent variation of seeding activity in three AD cases classified as low, moderate and high seeders in a previous study [15].

The relevance of FRET-based biosensor cells has been questioned by a study using recombinant tau fusion proteins [49]. According to the authors, when tau is fused to a fluorescent protein, steric hindrance may prevent the elongation of aggregates into fully formed filaments. Consequently, the FRET signal generated in biosensor cells may result from biological processes that diverge from aggregation, including stress granules or liquid–liquid phase separation. This study used a short 13–14 amino acid linker sequence between the tau fragment and the fluorescent reporter which is shorter than the linker present in the original biosensor line and the optimized sequence presented in this study. In addition, the dynamic quantification of aggregate formation in the biosensor cells using live cell imaging consistently followed a pattern composed by a lag phase, followed by a growth phase and a plateau (Supplementary Fig. 3) as previously reported in the original biosensor line [50]. This stereotypical pattern is reminiscent of models describing the kinetics of fibrillogenesis [51]. Here, we demonstrated that aggregates generated in biosensor cells after seeding consist of sarkosyl insoluble beta-pleated sheets. We have performed preliminary EM studies that failed to identify inclusions containing paired helical filaments in the cells. Thus, the FRET positive aggregates in this model should be viewed as evidence of seed competence of the tau presented to the cells, rather than as a model of PHF conformation per se. Recent elegant cryoEM studies [52] show conclusively that, although beta pleated sheet structures can be readily formed, even small changes in buffer conditions or in peptide length impact the likelihood of forming bone fide AD tau fibrils in vitro. Our current experience is aligned with these observations.

Here, we describe the methods for the design and generation of optimized FRET-based tau biosensor cell lines for the detection of tau seeding activity. While other methods such as RT-QuIC were proposed to quantify tau seeding activity, biosensor cells allow studies of templated aggregation in a cellular environment. The use of a cell line expressing LRP1, a known surface receptor for tau, allows studying uptake mechanisms and cellular downstream events using the same biosensor probe.

## Conclusions

The integration of the current knowledge of the structural basis of tau folds in AD and the role of post-translational modifications in the design of the probes led to an improved sensitivity and specificity. Thanks to the recent descriptions of tau fold in various tauopathies, such methods may guide the development of other biosensor lines designed for the detection of disease-specific conformers.

## Methods

### Cell culture

The Tau RD P301S FRET Biosensor cell line (CRL-3275) was provided by Marc Diamond. HEK293T cells were ordered from ATCC (CRL-11268). Cells were maintained in DMEM supplemented with 10% FBS and penicillin–streptomycin at 37 °C and 5% CO2 in a humidified atmosphere. CHO cell line was obtained from ATCC (CCL-61) and cultured in DMEM/F12 supplemented with 10% FBS and penicillin–streptomycin.

### Plasmids

Generation of the Tau-RD-P301S-CFP and Tau-RD-P301S-YFP lentiviral vectors has been described in [12]. Plasmids were provided by Marc Diamond. Fragments encoding for RD-P301S-CFP and RD-P301S-YFP were subcloned into a pcDNA3.1 plasmid containing a T2A self-cleaving peptide sequence. All other DNA constructs including the lentiviral vectors were synthesized and ordered from Twist Bioscience for expression plasmids or Vectorbuilder for lentiviral constructs. All synthesized DNA sequences were codon optimized using GeneDesign online tool [53]. The detailed map of the most efficient lentiviral construct and its sequence is provided in supplementary material.

### Human brain samples preparation

Frozen human cortical tissue was homogenized in PBS supplemented with 1X Halt protease inhibitor cocktail (ThermoFisher Scientific) (500 μl PBS for 100 mg tissue), with 20 up and down strokes in a 2 ml glass Dounce homogenizer. Samples were transferred in a 1.5 mL Eppendorf tube and centrifuged at 10,000 g for 10 min. Supernatant was collected, aliquoted and stored at -80 °C until further used. The high molecular weight (HMW) and low molecular weight (LMW) tau were prepared by size exclusion chromatography as described previously [21]. Briefly, brain homogenates were run on Superdex200 10/300GL columns (no. 17–5175-01, GE Healthcare) in PBS. Fractions of 500 μL were collected. HMW fractions 1 to 4 were pooled together and concentrated by ultracentrifugation at 150,000 g for 30 min at 4 °C. Supernatant was discarded and pellet was resuspended in 75 μL PBS to generate the HMW tau concentrate. The LMW fractions 12 to 14 were pooled together and concentrated approximately 20 times using an Ultra-15 (10 k) (Amicon, no. UFC901024) for 10 min at 4,000 g. Total tau concentration was quantified by western blot using serial dilution of tau-441 recombinant protein (Sigma-Aldrich no. T0576) and a rabbit polyclonal anti-human tau antibody (A0024, Dako) as primary antibody. The demographics and neuropathological diagnosis of the human cases that were used are summarized in Supplementary Table 2.

### Lentiviral transduction and clonal selection

Thirty thousand HEK293T cells per well were plated in a 24-well plate. After 24 h, cells were counted and infected with lentiviral particles with a multiplicity of infection of 30 in 250 μl conditioned medium supplemented with 5 μg/ml polybrene. The next day, 250 μL fresh culture medium was added. Cells were expanded for the following 2 weeks to allow for the elimination of infective viral particles. For clonal cell selections, cells were detached from a 75% confluent T75 culture flask with 5 ml Accutase and resuspended in 0.22 μm-filtered HBSS containing 1% bovine serum albumin. Cells were run on the Biorad S3e cell sorter and the 10% brightest cells were plated at 1 cell/well in 96-well plates containing 150 μL of 0.22 μm-filtered conditioned medium. Individual clones were cultured and amplified and characterized in seeding assays. The same procedure was followed for the transduction of CHO cells except that 10′000 CHO cells were plated in a 24-well plate for viral infection.

### Seeding assays

The tau seeding FRET-biosensor assay using transient transfection was performed as follows. HEK293T cells were reverse transfected in Costar Black (Corning) clear bottom 96-well plates (previously coated with 50 μg/mL poly-D-lysine), using 0.3 μL/well trans-IT X2 reagent (Mirus) in 10 μL/well Opti-MEM according to manufacturer’s protocol with 100 ng/well plasmid DNA. 6 × 10E5 cells were plated per well in a final volume of 100 μL. After 24 h, cells were washed with sterile PBS. Human brain extracts were incubated with Lipofectamine 2000 (Invitrogen, final concentration 1% vol/ vol) in Opti-MEM (final volume of 50 μL per well) for 20 min at room temperature before being added to the cells. Each condition was tested in triplicate or quadruplicate. For the seeding assay using the CRL-3275 cell line of stable 293 T clone, a similar procedure was used except that cells were not reverse transfected and that plate was seeded with 30,000 cells per well. Experiment was analyzed either by flow cytometry or live cell imaging on a Cytation 5 multimode reader (BioTek) with 1 image every 30 min. For flow cytometry, 24 h after incubation, with brain extracts, cells were collected with 50 μL trypsin, transferred into 96-well U-bottom plates (Corning) using 50 μL 10% FBS culture media to neutralize trypsin. Cells were pelleted at 1200 g for 10 min, resuspended in cold 2% paraformaldehyde for 10 min, pelleted at 1200 g, and resuspended in FACS buffer containing BSA, EDTA, and 0.09% azide (Miltenyi). Cells were analyzed on the MACSQuant VYB (Miltenyi) flow cytometer for the quantification of fluorescence and FRET. CFP/mTurquoise2 channel and FRET were both measured by exciting the cells using the 405-nm laser and reading fluorescence emission at the 405/50 nm and 525/50 nm filters, respectively. To quantify the FRET signal, a two-parameter density plot of FRET versus the CFP/mTurquoise2 donor was created, and cells treated with control or mock condition alone were used to identify the FRET-negative population. The percentage of FRET-positive cells and median fluorescence intensity was recorded. Integrated FRET density was calculated by multiplying the two parameters as proposed previously [14]. About 40,000 cells per well were analyzed. Flow cytometry data data was analyzed using FlowJo v10.7 software (BD Biosciences).

### Fluorescence imaging

For fluorescence imaging, seeding assays were performed in μClear film bottom 96-well black plates (Greiner Bio-One). The procedure was the same as described hereabove. All images were acquired on an Olympus FV3000 confocal laser scanning microscope. Cells were fixed in 4% PFA in PBS for 15 min. For immunocytochemistry, blocking was performed with 1% BSA, 0.1% Tween20 in PBS for 30 min. Primary antibody Tau-13 (Abcam) was applied overnight at 4 °C. For thiazine red staining, after fixation, cells were incubated for 15 min in 0.005% (w/v) solution of thiazine red in 50% ethanol in TBS solution and washed 3 times in TBS. Nuclear counterstaining was performed with 1 μg/mL DAPI solution for 5 min. To avoid overlap with mNeonGreen spectra, thiazine red was excited at 550 nm and recorded at 650 nm.

### Cell extracts, western blotting and immunodepeletion

For the analysis of tau reporter expression and solubility after seeding, cells were plated in 6-well plates (previously coated with 50 μg/mL poly-D-lysine) at a density of 9 × 10E5 cells per well. The next day, human brain extract (1 μg total tau per well) was incubated with Lipofectamine 2000 (Invitrogen, final concentration 1% vol/vol) in Opti-MEM (final volume of 2 mL per well) for 20 min at room temperature before being added to the cells. After 24 h, cells were washed 3 times with PBS and lysed in RIPA buffer containing 1X protease/phosphatase inhibitor cocktail. Total protein content was quantified by BCA. Volume of all samples was adjusted to match protein content. 1% sarkosyl final concentration was added and samples were incubated for 1 h at room temperature while shaking. Samples were then ultracentrifuged at 100,000 g for 1 h. The pellet was resuspended in PBS with 1X protease/phosphatase inhibitor cocktail. Western blots were run in 4–12% Bis–Tris gel (Invitrogen) in MOPS, transferred to PVDF membranes. Anti-tau 316–355 antibody targeting the repeat domain was used as a primary antibody (clone 77G7, BioLengend). Anti-GAPDH was used as loading control (Abcan, ab83956). Western blots were revealed on an Odyssey LI-COR system. For the immunodepleting of tau, anti-tau antibody (Cell Signaling Technology clone D5D8N) was cross-linked to protein G beads (5 μg of antibody for 25 μL of beads) and incubated in HMW tau sample (final volume 100 μL) overnight at 4 °C. Beads were pelleted by centrifugation and immunodepleted supernatant collected for analysis. Immunoprecipitation was confirmed by western blot on starting material and supernatant using a rabbit polyclonal anti-human tau antibody (A0024, Dako) as primary antibody.

### Statistics

Basic statistics and graphs were generated on GraphPad Prism v9.1.1 (GraphPad). *P* value was indicated as * for *p* < 0.05, ** for *p* < 0.01, *** for *p* < 0.001, **** for *p* < 0.0001. One-way ANOVA was used for all statistical analysis, except for Fig. 4D in which signal was compared using nonparametric Mann–Whitney test.

### Supplementary Information

**Additional file 1:** **Supplementary Table 1.** Calculated area under the curves for panels 1B, 1C and 1E. **Supplementary Figure 1.** Uncropped western blot membranes used to prepare figure 3B. A. Tau repeat domain primary antibody. B. GAPDH primary antibody. C. Original fluorescent image with the overlay of both channels. Samples were loaded in the following order: 1: CRL-3275 line transduced with lipofectamine only, soluble fraction, 2: CRL-3275 line transfected with lipofectamine only, insoluble fraction, 3: CRL-3275 line transfected with tau seeds, soluble fraction, 4: CRL-3275 line transfected with tau seeds, insoluble fraction, 5: HEK293T clone transfected with lipofectamine only, soluble fraction, 6: HEK293T clone transfected with lipofectamine only, insoluble fraction, 7: HEK293T clone transfected with tau seeds, soluble fraction, 8: HEK293T clone transfected with tau seeds, insoluble fraction. Other samples displayed on the membrane are not relevant for the present manuscript. D. Western blot quantification of tau repeat domain normalized on GAPDH expression in the CRL-3275 cells and HEK293T clone. **Supplementary Figure 2.** Detection of tau seeds in the absence of liposomes. Both CRL-3275 and HEK293T clone 18 were exposed to HMW or LMW AD brain-derived tau for 48 hours. The addition of 1 μM RAP reduced decreased seeding. **Supplementary Figure 3.** Kinetics of aggregate formation in novel biosensor clone 18. The novel HEK293T biosensor clone 18 was exposed to serial dilutions of AD brain lysate and run on a live cell imager for 60 hours. Aggregates were quantified every 30 minutes. Solid line is the mean of 4 replicates. Area is ±SEM for each point. **Supplementary Table 2.** Demographics and neuropathological diagnosis of human cases used for the study.

## Data Availability

The datasets used and/or analyzed during the current study are available from the corresponding author on reasonable request. The cell lines described in this manuscript will be deposited to the American Type Culture Collection.

## Abbreviations

MAPT: Microtubule associated protein tau
AD: Alzheimer’s disease
FRET: Förster resonance energy transfer
PHF: Paired helical filament
HEK: Human embryonic kidney
RD: Repeat domain
CFP: Cyan fluorescent protein
YFP: Yellow fluorescent protein
PSP: Progressive supranuclear palsy
CBD: Cortico basal degeneration
PiD: Pick’s disease
RT-QuIC: Real-time quaking induced conversion
EM: Electron microscopy
GAPDH: Glyceraldehyde-3-phosphate dehydrogenase
LRP1: LDL receptor related protein 1
HMW: High molecular weight
CHO: Chinese hamster ovary
