# Supplementary material for: Specific detection of tau seeding activity in Alzheimer’s disease using rationally designed biosensor cells
Source: Mol Neurodegener. 2023 Aug 8;18:53. doi: 10.1186/s13024-023-00643-2 (PMC10408046; doi:10.1186/s13024-023-00643-2)
Supplement: Supplementary file 1 — Additional file 1: Supplementary Table 1. Calculated area under the curves for panels 1B, 1C and 1E. Supplementary Figure 1. Uncropped western blot membranes used to prepare figure 3B. A. Tau repeat domain primary antibody. B. GAPDH primary antibody. C. Original fluorescent image with the overlay of both channels. Samples were loaded in the following order: 1: CRL-3275 line transduced with lipofectamine only, soluble fraction, 2: CRL-3275 line transfected with lipofectamine only, insoluble fraction, 3: CRL-3275 line transfected with tau seeds, soluble fraction, 4: CRL-3275 line transfected with tau seeds, insoluble fraction, 5: HEK293T clone transfected with lipofectamine only, soluble fraction, 6: HEK293T clone transfected with lipofectamine only, insoluble fraction, 7: HEK293T clone transfected with tau seeds, soluble fraction, 8: HEK293T clone transfected with tau seeds, insoluble fraction. Other samples displayed on the membrane are not relevant for the present manuscript. D. Western blot quantification of tau repeat domain normalized on GAPDH expression in the CRL-3275 cells and HEK293T clone. Supplementary Figure 2. Detection of tau seeds in the absence of liposomes. Both CRL-3275 and HEK293T clone 18 were exposed to HMW or LMW AD brain-derived tau for 48 hours. The addition of 1 μM RAP reduced decreased seeding. Supplementary Figure 3. Kinetics of aggregate formation in novel biosensor clone 18. The novel HEK293T biosensor clone 18 was exposed to serial dilutions of AD brain lysate and run on a live cell imager for 60 hours. Aggregates were quantified every 30 minutes. Solid line is the mean of 4 replicates. Area is ±SEM for each point. Supplementary Table 2. Demographics and neuropathological diagnosis of human cases used for the study. [file 13024_2023_643_MOESM1_ESM.pdf]

		AUC [%FRET-positive x pg]	CI 95%
Figure 1B	*Fusion*	2093	1360 to 2826
	(EAAAK) $_3$	3499	2549 to 4449
	(GGGGS) $_3$	49,31	0.000 to 102.5
	GGSGGSGG	301,1	165.8 to 436.3
	CRL-3275 construct	879,7	762.8 to 996.6
Figure 1C	*Fusion*	94556	61809 to 127302
	Fusion 3xKQ	338821	298021 to 379622
	(EAAAK) $_3$	116326	80231 to 152421
	(EAAAK) $_3$ *3xKQ*	468385	387640 to 549131
Figure 1E	*HEK293T (EAAAK)* $_3$ *3xKQ*	534984	473314 to 596653
	HEK293T Fusion 3xKQ	208033	170660 to 245407
	CRL-3275 cells	223364	181930 to 264798

Supplementary Table 1

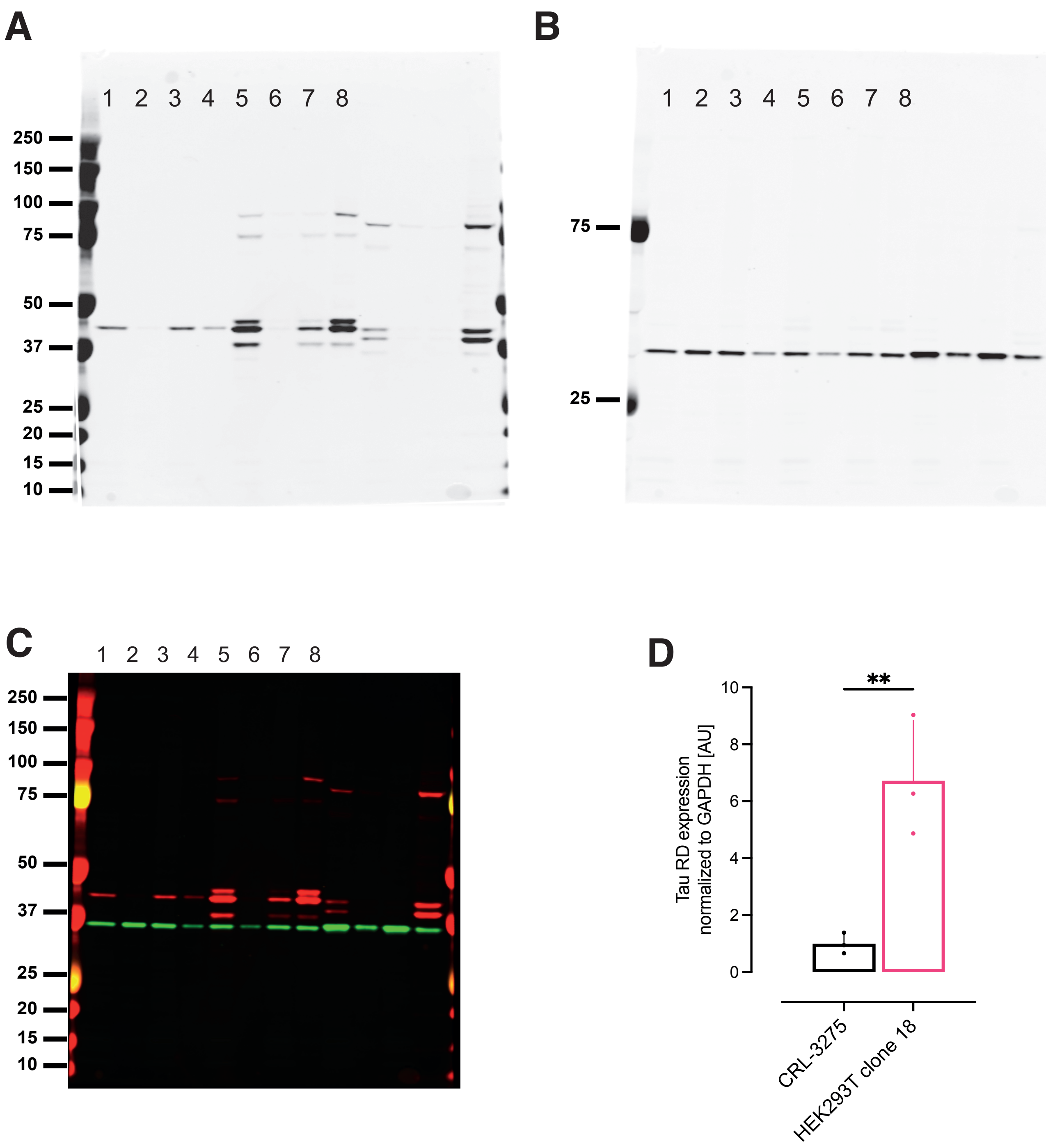

Supplementary Figure 1

A

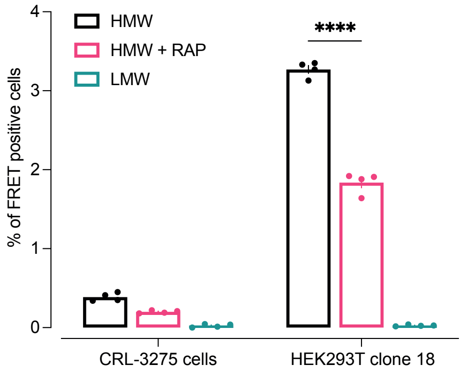

Supplementary Figure 2

A

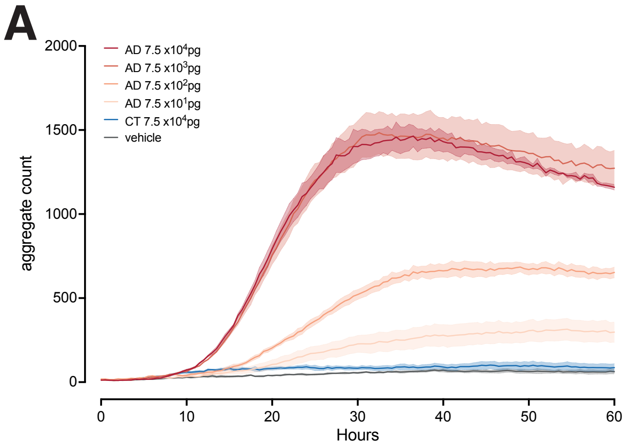

Supplementary Figure 3

Case	Age at Death	Sex	Neuropathological Diagnosis	Braak stage	Thal stage	ADNC A	ADNC B	ADNC C
AD1	61	Female	Alzheimer's Disease Neuropathological Changes (ADNC)	VI	5	3	3	3
AD2	77	Female	Alzheimer's Disease Neuropathological Changes (ADNC)	VI	5	3	3	3
AD3	77	Male	Alzheimer's Disease Neuropathological Changes (ADNC)	VI	5	3	3	3
AD4	91	Male	Alzheimer's Disease Neuropathological Changes (ADNC)	V	5	3	3	2
AD5	93	Male	Alzheimer's Disease Neuropathological Changes (ADNC)	IV	4	3	2	1
AD6	77	Female	Alzheimer's Disease Neuropathological Changes (ADNC)	IV	4	3	2	1
AD7	96	Female	Alzheimer's Disease Neuropathological Changes (ADNC)	IV	4	3	2	2
AD8	89	Male	Alzheimer's Disease Neuropathological Changes (ADNC)	IV	4	3	2	3
PSP1	72	Male	Progressive supranuclear palsy	NA	NA	NA	NA	NA
PSP2	77	Female	Progressive supranuclear palsy	NA	NA	NA	NA	NA
PSP3	71	Female	Progressive supranuclear palsy	NA	NA	NA	NA	NA
CBD1	70	Female	Cortico basal degeneration	NA	NA	NA	NA	NA
CBD2	64	Male	Cortico basal degeneration	NA	NA	NA	NA	NA
CBD3	63	Female	Cortico basal degeneration	NA	NA	NA	NA	NA
PiD1	77	Male	Pick's disease	NA	NA	NA	NA	NA
PiD2	72	Male	Pick's disease	NA	NA	NA	NA	NA
PiD3	71	Female	Pick's disease	NA	4	3	NA	2
CT1	73	Male	Control	0	2	1	0	1
CT2	63	Male	Control	0	2	1	0	0
CT3	56	Female	Control	0	1	1	0	0
Low seeder	73	Male	Alzheimer's Disease Neuropathological Changes (ADNC)	VI	4	3	3	3
Moderate seeder	82	Male	Alzheimer's Disease Neuropathological Changes (ADNC)	VI	5	3	3	2
High seeder	58	Female	Alzheimer's Disease Neuropathological Changes (ADNC)	VI	5	3	3	3

Supplementary Table 2

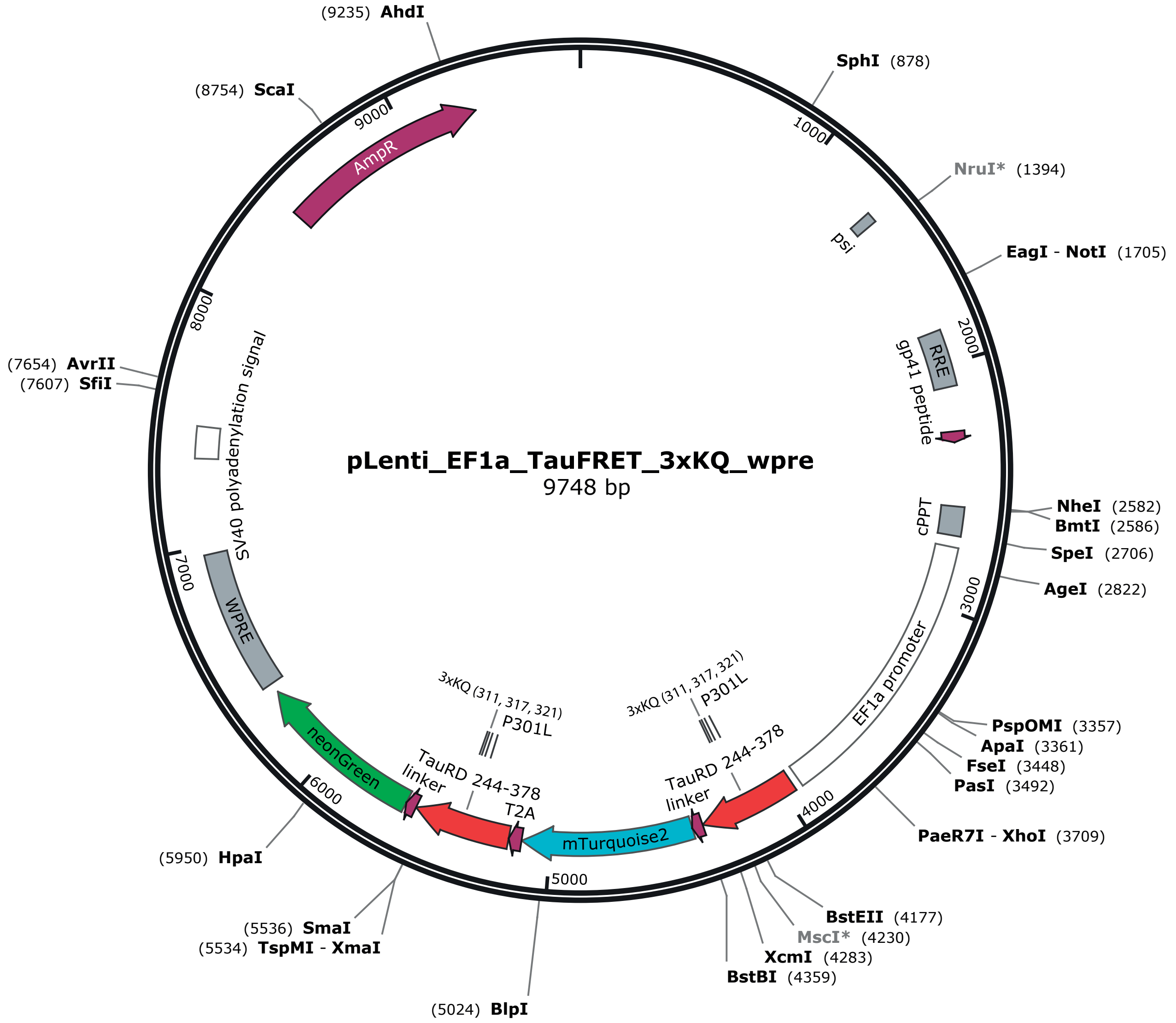

Supplementary Material

```
LOCUS       pLenti_EF1A_TauFRET_3xKQ_wpre        9748 bp    DNA
circular SYN 03-Sep-2020
DEFINITION  .
FEATURES             Location/Qualifiers
     regulatory      1306..1350
                     /regulatory_class="Other"
                     /label="psi"
                     /note="Retroviral Psi packaging element"
     regulatory      1860..2093
                     /regulatory_class="Other"
                     /label="RRE"
                     /note="Rev Response Element"
     CDS             2278..2322
                     /codon_start=1
                     /label="gp41 peptide"
                     /note="recognized by the 2H10 single-chain
llama nanobody"
                     /product="antigenic peptide corresponding to
amino acids
                     655 to 669 of the HIV envelope protein gp41 (<a
                     href=""https://www.ncbi.nlm.nih.gov/pubmed/
23505368"">Lutje
                     Hulsik et al., 2013)"
                     /transl_table=1
                     /translation="KNEQELLELDKWASL"
     misc_structure  2587..2706
                     /label="cPPT"
                     /note="Central Polypurine tract"
     regulatory      2754..3922
                     /note="EF1a promoter"
     CDS             3953..4360
                     /note="TauRD 244-378"
     modified_base   4127..4129
                     /note="P301L"
     modified_base   4157..4159
                     /note="K311Q"
     modified_base   4175..4177
                     /note="K317Q"
     modified_base   4187..4189
                     /note="K321Q"
     CDS             4361..4405
                     /note="linker"
     CDS             4406..5119
                     /note="mTurquoise2"
     CDS             5120..5173
                     /note="T2A"
     CDS             5174..5578
                     /note="TauRD 244-378"
     modified_base   5345..5347
                     /note="P301L"
     modified_base   5375..5377
                     /note="K311Q"
     modified_base   5393..5395
                     /note="K317Q"
```

```
     modified_base   5405..5407
                     /note="K321Q"
     CDS             5579..5623
                     /note="linker"
     CDS             5624..6331
                     /note="neonGreen"
     regulatory      6374..6966
                     /regulatory_class="Other"
                     /label="WPRE"
                     /note="Woodchuck Hepatitis Virus
Posttranscriptional
                     Regulatory Element"
     regulatory      7359..7489
                     /regulatory_class="polyA_signal_sequence"
                     /label="SV40 pA"
                     /note="SV40 polyadenylation signal"
     CDS             8448..9308
                     /codon_start=1
                     /db_xref="GI:455370"
                     /gene="bla"
                     /label="AmpR"
                     /note="E-286"
                     /product="beta-lactamase"
                     /protein_id="AAB59737.1"
                     /transl_table=11
                     /
translation="MSIQHFRVALIPFFAAFCLPVFAHPETLVKVKDAEDQLGARVGYI

ELDLNSGKILESFRPEERFPMMSTFKVLLCGAVLSRVDAGQEQLGRRIHYSQNDLVEYS

PVTEKHLTDGMTVRELCSAAITMSDNTAANLLLTTIGGPKELTAFLHNMGDHVTRLDRW

EPELNEAIPNDERDTTMPAAMATTLRKLLTGELLTLASRQQLIDWMEADKVAGPLLRSA

LPAGWFIADKSGAGERGSRGIIAALGPDGKPSRIVVIYTTGSQATMDERNRQIAEIGAS
                     LIKHW"
BASE COUNT  2496 A  2408 C  2580 G  2264 T  0 OTHER
ORIGIN
        1 GTTGGACTCA AGACGATAGT TACCGGATAA GGCGCAGCGG TCGGGCTGAA
CGGGGGGTTC
       61 GTGCACACAG CCCAGCTTGG AGCGAACGAC CTACACCGAA CTGAGATACC
TACAGCGTGA
      121 GCTATGAGAA AGCGCCACGC TTCCCGAAGA GAGAAAGGCG GACAGGTATC
CGGTAAGCGG
      181 CAGGGTCGGA ACAGGAGAGC GCACGAGGGA GCTTCCAGGG GGAAACGCCT
GGTATCTTTA
      241 TAGTCCTGTC GGGTTTCGCC ACCTCTGACT TGAGCGTCGA TTTTTGTGAT
GCTCGTCAGG
      301 GGGGCGGAGC CTATGGAAAA ACGCCAGCAA CGCGGCCTTT TTACGGTTCC
TGGCCTTTTG
      361 CTGGCCTTTT GCTCACATGT TCTTTCCTGC GTTATCCCCT GATTCTGTGG
ATAACCGTAT
      421 TACCGCCTTT GAGTGAGCTG ATACCGCTCG CCGCAGCCGA ACGACCGAGC
GCAGCGAGTC
```

481 AGTGAGCGAG GAAGCGGAAG AGCGCCCAAT ACGCAAACCG CCTCTCCCCG
CGCGTTGGCC
541 GATTCATTAA TGCAGCTGGC ACGACAGGTT TCCCGACTGG AAAGCGGGCA
GTGAGCGCAA
601 CGCAATTAAT GTGAGTTAGC TCACTCATTA GGCACCCCAG GCTTTACACT
TTATGCTTCC
661 GGCTCGTATG TTGTGTGGAA TTGTGAGCGG ATAACAATTT CACACAGGAA
ACAGCTATGA
721 CCATGATTAC GCCAAGCGCG CAATTAACCC TCACTAAAGG GAACAAAAGC
TGGAGCTGCA
781 AGCTTAATGT AGTCTTATGC AATACTCTTG TAGTCTTGCA ACATGGTAAC
GATGAGTTAG
841 CAACATGCCT TACAAGGAGA GAAAAAGCAC CGTGCATGCC GATTGGTGGA
AGTAAGGTGG
901 TACGATCGTG CCTTATTAGG AAGGCAACAG ACGGGTCTGA CATGGATTGG
ACGAACCACT
961 GAATTGCCGC ATTGCAGAGA TATTGTATTT AAGTGCCTAG CTCGATACAT
AAACGGGTCT
1021 CTCTGGTTAG ACCAGATCTG AGCCTGGGAG CTCTCTGGCT AACTAGGGAA
CCCACTGCTT
1081 AAGCCTCAAT AAAGCTTGCC TTGAGTGCTT CAAGTAGTGT GTGCCCGTCT
GTTGTGTGAC
1141 TCTGGTAACT AGAGATCCCT CAGACCCTTT TAGTCAGTGT GGAAAATCTC
TAGCAGTGGC
1201 GCCCGAACAG GGACTTGAAA GCGAAAGGGA AACCAGAGGA GCTCTCTCGA
CGCAGGACTC
1261 GGCTTGCTGA AGCGCGCACG GCAAGAGGCG AGGGGCGGCG ACTGGTGAGT
ACGCCAAAAA
1321 TTTTGACTAG CGGAGGCTAG AAGGAGAGAG ATGGGTGCGA GAGCGTCAGT
ATTAAGCGGG
1381 GGAGAATTAG ATCGCGATGG GAAAAAATTC GGTTAAGGCC AGGGGGAAAG
AAAAAATATA
1441 AATTAAAACA TATAGTATGG GCAAGCAGGG AGCTAGAACG ATTCGCAGTT
AATCCTGGCC
1501 TGTTAGAAAC ATCAGAAGGC TGTAGACAAA TACTGGGACA GCTACAACCA
TCCCTTCAGA
1561 CAGGATCAGA AGAACTTAGA TCATTATATA ATACAGTAGC AACCCTCTAT
TGTGTGCATC
1621 AAAGGATAGA GATAAAAGAC ACCAAGGAAG CTTTAGACAA GATAGAGGAA
GAGCAAAACA
1681 AAAGTAAGAC CACCGCACAG CAAGCGGCCG CTGATCTTCA GACCTGGAGG
AGGAGATATG
1741 AGGGACAATT GGAGAAGTGA ATTATATAAA TATAAAGTAG TAAAAATTGA
ACCATTAGGA
1801 GTAGCACCCA CCAAGGCAAA GAGAAGAGTG GTGCAGAGAG AAAAAAGAGC
AGTGGGAATA
1861 GGAGCTTTGT TCCTTGGGTT CTTGGGAGCA GCAGGAAGCA CTATGGGCGC
AGCGTCAATG
1921 ACGCTGACGG TACAGGCCAG ACAATTATTG TCTGGTATAG TGCAGCAGCA
GAACAATTTG
1981 CTGAGGGCTA TTGAGGCGCA ACAGCATCTG TTGCAACTCA CAGTCTGGGG
CATCAAGCAG
2041 CTCCAGGCAA GAATCCTGGC TGTGGAAAGA TACCTAAAGG ATCAACAGCT
CCTGGGGATT

2101 TGGGGTTGCT CTGGAAAACT CATTTGCACC ACTGCTGTGC CTTGGAATGC TAGTTGGAGT
2161 AATAAATCTC TGGAACAGAT TTGGAATCAC ACGACCTGGA TGGAGTGGGA CAGAGAAATT
2221 AACAATTACA CAAGCTTAAT ACACTCCTTA ATTGAAGAAT CGCAAAACCA GCAAGAAAAG
2281 AATGAACAAG AATTATTGGA ATTAGATAAA TGGGCAAGTT TGTGGAATTG GTTTAACATA
2341 ACAAATTGGC TGTGGTATAT AAAATTATTC ATAATGATAG TAGGAGGCTT GGTAGGTTTA
2401 AGAATAGTTT TTGCTGTACT TTCTATAGTG AATAGAGTTA GGCAGGGATA TTCACCATTA
2461 TCGTTTCAGA CCCACCTCCC AACCCCGAGG GGACCCGACA GGCCCGAAGG AATAGAAGAA
2521 GAAGGTGGAG AGAGAGACAG AGACAGATCC ATTCGATTAG TGAACGGATC TCGACGGTAT
2581 CGCTAGCTTT TAAAAGAAAA GGGGGGATTG GGGGGTACAG TGCAGGGGAA AGAATAGTAG
2641 ACATAATAGC AACAGACATA CAAACTAAAG AATTACAAAA ACAAATTACA AAAATTCAAA
2701 ATTTTACTAG TGATTATCGG ATCAACTTTG TATAGAAAAG TTGGGCTCCG GTGCCCGTCA
2761 GTGGGCAGAG CGCACATCGC CCACAGTCCC CGAGAAGTTG GGGGGAGGGG TCGGCAATTG
2821 AACCGGTGCC TAGAGAAGGT GGCGCGGGGT AAACTGGGAA AGTGATGTCG TGTACTGGCT
2881 CCGCCTTTTT CCCGAGGGTG GGGGAGAACC GTATATAAGT GCAGTAGTCG CCGTGAACGT
2941 TCTTTTTCGC AACGGGTTTG CCGCCAGAAC ACAGGTAAGT GCCGTGTGTG GTTCCCGCGG
3001 GCCTGGCCTC TTTACGGGTT ATGGCCCTTG CGTGCCTTGA ATTACTTCCA CCTGGCTGCA
3061 GTACGTGATT CTTGATCCCG AGCTTCGGGT TGGAAGTGGG TGGGAGAGTT CGAGGCCTTG
3121 CGCTTAAGGA GCCCCTTCGC CTCGTGCTTG AGTTGAGGCC TGGCCTGGGC GCTGGGGCCG
3181 CCGCGTGCGA ATCTGGTGGC ACCTTCGCGC CTGTCTCGCT GCTTTCGATA AGTCTCTAGC
3241 CATTTAAAAT TTTTGATGAC CTGCTGCGAC GCTTTTTTTC TGGCAAGATA GTCTTGTAAA
3301 TGCGGGCCAA GATCTGCACA CTGGTATTTC GGTTTTTGGG GCCGCGGGCG GCGACGGGGC
3361 CCGTGCGTCC CAGCGCACAT GTTCGGCGAG GCGGGGCCTG CGAGCGCGGC CACCGAGAAT
3421 CGGACGGGGG TAGTCTCAAG CTGGCCGGCC TGCTCTGGTG CCTGGTCTCG CGCCGCCGTG
3481 TATCGCCCCG CCCTGGGCGG CAAGGCTGGC CCGGTCGGCA CCAGTTGCGT GAGCGGAAAG
3541 ATGGCCGCTT CCCGGCCCTG CTGCAGGGAG CTCAAAATGG AGGACGCGGC GCTCGGGAGA
3601 GCGGGCGGGT GAGTCACCCA CACAAAGGAA AAGGGCCTTT CCGTCCTCAG CCGTCGCTTC
3661 ATGTGACTCC ACGGAGTACC GGGCGCCGTC CAGGCACCTC GATTAGTTCT CGAGCTTTTG

3721 GAGTACGTCG TCTTTAGGTT GGGGGGAGGG GTTTTATGCG ATGGAGTTTC CCCACACTGA
3781 GTGGGTGGAG ACTGAAGTTA GGCCAGCTTG GCACTTGATG TAATTCTCCT TGGAATTTGC
3841 CCTTTTTGAG TTTGGATCTT GGTTCATTCT CAAGCCTCAG ACAGTGGTTC AAAGTTTTTT
3901 TCTTCCATTT CAGGTGTCGT GACAAGTTTG TACAAAAAAG CAGGCTGCCA CCATGCAGAC
3961 AGCCCCTGTG CCCATGCCAG ACCTGAAGAA TGTCAAATCC AAGATCGGCT CCACTGAGAA
4021 CCTGAAGCAC CAGCCGGGAG GCGGGAAGGT GCAGATAATT AATAAGAAGC TGGATCTTAG
4081 CAACGTCCAG TCCAAATGCG GATCAAAGGA TAATATCAAA CACGTCTTGG GAGGCGGCAG
4141 TGTGCAAATA GTCTACCAAC CAGTTGACCT GAGCCAGGTG ACCTCCCAGT GTGGCTCATT
4201 AGGCAACATC CATCATAAAC CAGGAGGTGG CCAGGTGGAA GTAAAATCTG AGAAGCTTGA
4261 CTTCAAGGAC AGAGTCCAGT CGAAGATTGG GTCCCTGGAC AATATCACCC ACGTCCCTGG
4321 CGGAGGAAAT AAAAAGATTG AAACCCACAA GCTGACCTTC GAAGCAGCCG CTAAAGAGGC
4381 CGCTGCGAAG GAAGCCGCAG CAAAAGTGAG CAAGGGAGAA GAGCTGTTCA CCGGGGTGGT
4441 GCCCATCCTG GTCGAGCTGG ACGGCGACGT AAACGGCCAC AAGTTCAGCG TGTCCGGCGA
4501 GGGCGAGGGC GATGCCACCT ACGGCAAGCT GACCCTGAAG TTCATCTGCA CCACCGGCAA
4561 GCTGCCCGTG CCCTGGCCCA CACTCGTGAC CACCCTGTCC TGGGGCGTGC AGTGCTTCGC
4621 CCGCTACCCC GACCACATGA AGCAGCACGA CTTCTTCAAG TCCGCCATGC CCGAAGGCTA
4681 CGTCCAGGAG CGCACCATCT TCTTCAAGGA CGACGGCAAC TACAAGACCC GCGCCGAGGT
4741 GAAGTTCGAG GGCGACACCC TGGTGAACCG CATCGAGCTG AAGGGCATCG ACTTCAAGGA
4801 GGACGGCAAC ATCCTGGGGC ACAAGCTGGA GTACAACTAC TTTAGCGACA ACGTCTATAT
4861 CACCGCCGAC AAGCAGAAGA ACGGCATCAA GGCCAACTTC AAGATCCGCC ACAACATCGA
4921 GGACGGCGGC GTGCAGCTCG CCGACCACTA CCAGCAGAAC ACCCCCATCG GCGACGGCCC
4981 CGTGCTGCTG CCCGACAACC ACTACCTGAG CACCCAGTCC AAGCTGAGCA AAGACCCCAA
5041 CGAGAAGCGC GATCACATGG TCCTGCTGGA GTTCGTGACC GCCGCCGGGA TCACTCTCGG
5101 AATGGATGAA CTCTACAAAG AGGGCAGAGG AAGTCTGCTA ACATGCGGTG ACGTCGAGGA
5161 GAATCCTGGA CCTCAAACCG CGCCCGTCCC GATGCCCGAT CTGAAAAACG TGAAAAGCAA
5221 GATTGGGAGC ACCGAGAATC TGAAACATCA ACCCGGCGGG GGCAAAGTCC AGATCATCAA
5281 CAAGAAACTC GACCTGTCCA ATGTGCAAAG CAAGTGTGGG AGCAAAGACA ACATCAAGCA

5341 TGTGCTGGGC GGGGGGAGCG TCCAGATCGT GTATCAGCCC GTGGATCTCT CCCAGGTCAC
5401 GAGCCAATGC GGCAGCCTGG GCAATATCCA CCATAAGCCC GGCGGCGGGC AAGTCGAGGT
5461 GAAGAGCGAA AAACTGGATT TTAAGGACCG CGTGCAAAGC AAAATCGGCA GCCTCGACAA
5521 CATTACGCAT GTGCCCGGGG GCGGCAACAA GAAGATCGAG ACCCATAAAC TCACCTTTGA
5581 GGCCGCCGCA AAGGAAGCTG CAGCCAAAGA GGCCGCCGCC AAGGTCTCCA AAGGCGAGGA
5641 AGATAACATG GCATCTCTAC CAGCAACACA TGAGTTACAC ATATTTGGGT CCATCAATGG
5701 AGTCGATTTT GACATGGTGG GGCAGGGAAC AGGCAATCCA AATGATGGTT ATGAGGAATT
5761 GAATTTGAAG TCCACAAAAG GAGACCTCCA GTTCTCACCC TGGATTCTGG TCCCTCATAT
5821 TGGGTATGGC TTCCACCAGT ATCTGCCCTA CCCTGACGGG ATGAGCCCTT TCCAGGCAGC
5881 CATGGTAGAT GGCTCTGGCT ACCAAGTCCA TCGAACAATG CAGTTTGAAG ATGGTGCCTC
5941 CCTTACTGTT AACTACCGCT ATACCTACGA GGGAAGCCAC ATCAAAGGAG AGGCCCAGGT
6001 GAAAGGGACT GGTTTCCCCG CTGACGGTCC TGTGATGACC AACTCACTGA CCGCTGCTGA
6061 CTGGTGCAGG TCGAAAAAGA CTTATCCCAA TGACAAAACA ATTATCAGTA CCTTTAAGTG
6121 GAGTTACACC ACTGGAAATG GAAAGAGATA CCGGAGCACT GCCAGAACCA CGTACACCTT
6181 TGCCAAGCCA ATGGCGGCTA ACTATCTGAA GAACCAGCCG ATGTACGTGT TCCGTAAGAC
6241 GGAGCTTAAG CACTCTAAAA CCGAACTCAA TTTCAAAGAA TGGCAAAAGG CCTTTACAGA
6301 TGTTATGGGC ATGGACGAGC TGTATAAGTA AGTAAACCCA GCTTTCTTGT ACAAAGTGGT
6361 GATAATCGAA TTCCGATAAT CAACCTCTGG ATTACAAAAT TTGTGAAAGA TTGACTGGTA
6421 TTCTTAACTA TGTTGCTCCT TTTACGCTAT GTGGATACGC TGCTTTAATG CCTTTGTATC
6481 ATGCTATTGC TTCCCGTATG GCTTTCATTT TCTCCTCCTT GTATAAATCC TGGTTGCTGT
6541 CTCTTTATGA GGAGTTGTGG CCCGTTGTCA GGCAACGTGG CGTGGTGTGC ACTGTGTTTG
6601 CTGACGCAAC CCCCACTGGT TGGGGCATTG CCACCACCTG TCAGCTCCTT TCCGGGACTT
6661 TCGCTTTCCC CCTCCCTATT GCCACGGCGG AACTCATCGC CGCCTGCCTT GCCCGCTGCT
6721 GGACAGGGGC TCGGCTGTTG GGCACTGACA ATTCCGTGGT GTTGTCGGGG AAGCTGACGT
6781 CCTTTCCATG GCTGCTCGCC TGTGTTGCCA CCTGGATTCT GCGCGGGACG TCCTTCTGCT
6841 ACGTCCCTTC GGCCCTCAAT CCAGCGGACC TTCCTTCCCG CGGCCTGCTG CCGGCTCTGC
6901 GGCCTCTTCC GCGTCTTCGC CTTCGCCCTC AGACGAGTCG GATCTCCCTT TGGGCCGCCT

6961 CCCCGCATCG GGAATTCCCG CGGTTCGCTT TAAGACCAAT GACTTACAAG GCAGCTGTAG
7021 ATCTTAGCCA CTTTTTAAAA GAAAAGGGGG GACTGGAAGG GCTAATTCAC TCCCAACGAA
7081 GACAAGATCT GCTTTTTGCT TGTACTGGGT CTCTCTGGTT AGACCAGATC TGAGCCTGGG
7141 AGCTCTCTGG CTAACTAGGG AACCCACTGC TTAAGCCTCA ATAAAGCTTG CCTTGAGTGC
7201 TTCAAGTAGT GTGTGCCCGT CTGTTGTGTG ACTCTGGTAA CTAGAGATCC CTCAGACCCT
7261 TTTAGTCAGT GTGGAAAATC TCTAGCAGTA GTAGTTCATG TCATCTTATT ATTCAGTATT
7321 TATAACTTGC AAAGAAATGA ATATCAGAGA GTGAGAGGAA CTTGTTTATT GCAGCTTATA
7381 ATGGTTACAA ATAAAGCAAT AGCATCACAA ATTTCACAAA TAAAGCATTT TTTTCACTGC
7441 ATTCTAGTTG TGGTTTGTCC AAACTCATCA ATGTATCTTA TCATGTCTGG CTCTAGCTAT
7501 CCCGCCCCTA ACTCCGCCCA TCCCGCCCCT AACTCCGCCC AGTTCCGCCC ATTCTCCGCC
7561 CCATGGCTGA CTAATTTTTT TTATTTATGC AGAGGCCGAG GCCGCCTCGG CCTCTGAGCT
7621 ATTCCAGAAG TAGTGAGGAG GCTTTTTTGG AGGCCTAGGG ACGTACCCAA TTCGCCCTAT
7681 AGTGAGTCGT ATTACGCGCG CTCACTGGCC GTCGTTTTAC AACGTCGTGA CTGGGAAAAC
7741 CCTGGCGTTA CCCAACTTAA TCGCCTTGCA GCACATCCCC CTTTCGCCAG CTGGCGTAAT
7801 AGCGAAGAGG CCCGCACCGA TCGCCCTTCC CAACAGTTGC GCAGCCTGAA TGGCGAATGG
7861 GACGCGCCCT GTAGCGGCGC ATTAAGCGCG GCGGGTGTGG TGGTTACGCG CAGCGTGACC
7921 GCTACACTTG CCAGCGCCCT AGCGCCCGCT CCTTTCGCTT TCTTCCCTTC CTTTCTCGCC
7981 ACGTTCGCCG GCTTTCCCCG TCAAGCTCTA AATCGGGGGC TCCCTTTAGG GTTCCGATTT
8041 AGTGCTTTAC GGCACCTCGA CCCCAAAAAA CTTGATTAGG GTGATGGTTC ACGTAGTGGG
8101 CCATCGCCCT GATAGACGGT TTTTCGCCCT TTGACGTTGG AGTCCACGTT CTTTAATAGT
8161 GGACTCTTGT TCCAAACTGG AACAACACTC AACCCTATCT CGGTCTATTC TTTTGATTTA
8221 TAAGGGATTT TGCCGATTTC GGCCTATTGG TTAAAAAATG AGCTGATTTA ACAAAAATTT
8281 AACGCGAATT TTAACAAAAT ATTAACGCTT ACAATTTAGG TGGCACTTTT CGGGGAAATG
8341 TGCGCGGAAC CCCTATTTGT TTATTTTTCT AAATACATTC AAATATGTAT CCGCTCATGA
8401 GACAATAACC CTGATAAATG CTTCAATAAT ATTGAAAAAG GAAGAGTATG AGTATTCAAC
8461 ATTTCCGTGT CGCCCTTATT CCCTTTTTTG CGGCATTTTG CCTTCCTGTT TTTGCTCACC
8521 CAGAAACGCT GGTGAAAGTA AAAGATGCTG AAGATCAGTT GGGTGCACGA GTGGGTTACA

8581 TCGAACTGGA TCTCAACAGC GGTAAGATCC TTGAGAGTTT TCGCCCCGAA GAACGTTTTC
8641 CAATGATGAG CACTTTTAAA GTTCTGCTAT GTGGCGCGGT ATTATCCCGT ATTGACGCCG
8701 GGCAAGAGCA ACTCGGTCGC CGCATACACT ATTCTCAGAA TGACTTGGTT GAGTACTCAC
8761 CAGTCACAGA AAAGCATCTT ACGGATGGCA TGACAGTAAG AGAATTATGC AGTGCTGCCA
8821 TAACCATGAG TGATAACACT GCGGCCAACT TACTTCTGAC AACGATCGGA GGACCGAAGG
8881 AGCTAACCGC TTTTTTGCAC AACATGGGGG ATCATGTAAC TCGCCTTGAT CGTTGGGAAC
8941 CGGAGCTGAA TGAAGCCATA CCAAACGACG AGCGTGACAC CACGATGCCT GTAGCAATGG
9001 CAACAACGTT GCGCAAACTA TTAACTGGCG AACTACTTAC TCTAGCTTCC CGGCAACAAT
9061 TAATAGACTG GATGGAGGCG GATAAAGTTG CAGGACCACT TCTGCGCTCG GCCCTTCCGG
9121 CTGGCTGGTT TATTGCTGAT AAATCTGGAG CCGGTGAGCG TGGGTCTCGC GGTATCATTG
9181 CAGCACTGGG GCCAGATGGT AAGCCCTCCC GTATCGTAGT TATCTACACG ACGGGGAGTC
9241 AGGCAACTAT GGATGAACGA AATAGACAGA TCGCTGAGAT AGGTGCCTCA CTGATTAAGC
9301 ATTGGTAACT GTCAGACCAA GTTTACTCAT ATATACTTTA GATTGATTTA AAACTTCATT
9361 TTTAATTTAA AAGGATCTAG GTGAAGATCC TTTTTGATAA TCTCATGACC AAAATCCCTT
9421 AACGTGAGTT TTCGTTCCAC TGAGCGTCAG ACCCCGTAGA AAAGATCAAA GGATCTTCTT
9481 GAGATCCTTT TTTTCTGCGC GTAATCTGCT GCTTGCAAAC AAAAAAACCA CCGCTACCAG
9541 CGGTGGTTTG TTTGCCGGAT CAAGAGCTAC CAACTCTTTT TCCGAAGGTA ACTGGCTTCA
9601 GCAGAGCGCA GATACCAAAT ACTGTTCTTC TAGTGTAGCC GTAGTTAGGC CACCACTTCA
9661 AGAACTCTGT AGCACCGCCT ACATACCTCG CTCTGCTAAT CCTGTTACCA GTGGCTGCTG
9721 CCAGTGGCGA TAAGTCGTGT CTTACCGG
//
